# Advances and Challenges in Islet Transplantation: Islet Procurement Rates and Lessons Learned from Suboptimal Islet Transplantation

**DOI:** 10.1155/2011/979527

**Published:** 2011-12-22

**Authors:** Annette Plesner, C. Bruce Verchere

**Affiliations:** Department of Pathology and Laboratory Medicine, Child and Family Research Institute, The University of British Columbia, 950 West 28th Avenue, Vancouver, BC, Canada V5Z 4H4

## Abstract

The initial step in successful islet transplantation is procurement of healthy donor islets. Given the limited number of donor pancreata selected for islet isolation and that islets from multiple donors are typically required to obtain insulin independence, it is critical to improve pancreas procurement rates and yield of islets for transplantation. Islets are delicate microorgans that are susceptible to apoptosis, hypoxia, and ischemia during isolation, culture, and the peritransplant period. Once the islets are engrafted, both prompt revascularization and protection from beta-cell death and graft rejection are key to secure long-term survival and function. To facilitate the engraftment of more robust islets suitable for combating the challenging isolation period and proinflammatory transplantation milieu, numerous approaches have been employed to prevent beta-cell dysfunction and death including immune modulation, prevention of apoptosis and hypoxia, as well as stimulation of growth factors, angiogenesis, and reinnervation. In addition to briefly discussing islet isolation procedures, procurement rates, and islet transplantation, the relevant literature pertaining to successful suboptimal islet transplantation is reviewed to provide insight into potential approaches to balance the limited supply of available donor islets.

## 1. Islet Transplantation

Islet transplantation is an experimental procedure available to a limited group of type 1 diabetes patients. The procedure was pioneered by Lacy in 1967, when he established a collagenase-based isolation procedure to procure islets from rat pancreata [1]. A few years later, Lacy and colleagues reported the first successful islet transplantation in rodents and primates [2, 3], and by the late 1980s the first islet transplant to obtain insulin independence in a diabetic patient was achieved [4]. Over the next decade, optimization of the islet isolation protocol and use of immunosuppressive drugs with less deleterious side effects became focal points in the field culminating with the establishment of the Edmonton protocol in 2000 [5, 6]. The success of the Edmonton group arose in part because of the use of freshly isolated islets from multiple donors, xenoprotein-free culture conditions, and omitting the use of corticosteroids to prevent rejection. Instead, a combination of immunosuppressive drugs was used that targets IL-2 and hence T- and B-cell stimulation to prevent islet allograft rejection and diminish recurrent autoimmunity. The standard immunosuppressive cocktail used comprises tacrolimus (a calcineurin inhibitor that blocks IL-2 production), sirolimus (inhibitor of the mTOR protein kinase involved in signal transduction and lymphocyte proliferation), and anti-IL2 receptor antibodies (diminishes IL-2 driven T-cell proliferation during the acute rejection phase).

Obtaining glucose stability is crucial to diabetes patients with acquisition of insulin independence being the obvious long-term goal. Longitudinal studies have shown that 75% of islet grafts fail within the first two years after transplantation, and hence patients return to exogenous insulin therapy [7]. To evaluate the success of islet transplantation, a beta score has been established as a physiological measure of beta-cell function that simultaneously evaluates glycemic control, endogenous insulin secretion, and diabetes therapy [8]. The beta score has been found to be inversely proportional to the level of panel reactivity antibodies such that a high level of panel reactive antibodies is associated with a lower beta score that is indicative of a decreased islet transplantation success rate and vice versa [9]. Even in transplant recipients that return to insulin therapy, the insulin dose required is typically lower than that used before transplant and the islet graft ensures that the patient largely avoids the potentially life-threatening hypoglycemic episodes associated with insulin therapy. In conjunction with actual beta-cell loss, beta-cell dysfunction also seems to contribute to insulin dependence given that most transplant recipients have residual serum C-peptide [10–12]. Data suggest that ~50–70% of the transplanted islet cells undergo apoptosis during isolation, culture, and the peritransplant period [13]. Numerous challenges remain in order to move the field ahead: optimize the isolation procedure to improve islet yield, purity, and function, optimize culture conditions to improve the quality of pretransplant material, and improve posttransplant graft survival by fine-tuning the immunosuppression regimen to be less diabetogenic [14–16]. Due to the vicinity of the endocrine tissue to digestive enzymes in the pancreas, fast procurement post-mortem is necessary. Apart from mechanical damage during the islet isolation procedure, islets are separated from their nourishing microenvironment and subjected to devascularization, denervation, and hypoxia [17]. The brief culture period after isolation may provide the islet with a much-needed recovery period prior to transplantation and may also allow for depletion of passenger leukocytes and deactivation of intracellular stress signaling pathways to diminish allorejection [18]. Once transplanted, islets need to revascularize and reinnervate rapidly for survival and proper glucose sensing but at this critical time point also face allorejection, recurrent autoimmunity, possible amyloid deposition, and metabolic stress. Delayed angiogenesis, immune and nonimmune mechanisms that result in the release of cytokines, chemokines, and reactive oxygen species and ensuing ER stress all counter successful islet engraftment and function. The immunosuppressive regimens in current use have known toxicity to islet cells, with tacrolimus and sirolimus showing deleterious effects on both duct and beta-cell growth and survival as well as causing nephrotoxicity [19, 20]. An additional side effect of sirolimus reported in female patients after transplant is the occurrence of ovarian cysts [21]. Lifelong immune suppression increases the susceptibility to opportunistic infections and incidence of malignancy [22].

Non-immune mechanisms partake in islet graft failure, evident from a proportion of islet autograft recipients who still require insulin therapy following transplant [23–25]. However, given that the 5-year insulin-independence requirement for autografts is similar to the 2-year allograft persistence rate, autografts appear to be more durable, likely due to the lack of allo- and autoimmune rejection as well as the lack of any potentially toxic effect of immune suppression [9]. Indeed a recent study demonstrated that insulin secretion and glucose tolerance were similar in recipients of an islet allograft and autograft only when the allograft group received double the number of islets [24]. In addition, the contribution of a heterotopic transplant site and ensuing alternative implant microenvironment should not be underestimated despite the less invasive nature of infusing islets into the portal vein compared to whole-organ pancreas transplantation [26]. Ultimately it is of importance to the islet transplantation field to optimize the islet isolation protocol as well as minimize the various destabilizing or toxic contributors to the graft environment, since multiple factors reduce the effective number of functional islets after transplantation. To this end several recent promising advances have been made to optimize the field: one study persufflated the pancreas to increase oxygenation of the tissue which elevates ATP levels during preservation [27]; another study targeted donor-specific memory T cells with a neutralizing antibody to the adhesion molecule lymphocyte function-associated antigen 1 in combination with a cocktail of basiliximab and sirolimus or belatacept in order to minimize the activity of this blockade-resistant population of memory T cells [28].

## 2. Islet Yield and Recovery in Mice and Men

The standard donor-to-recipient ratio for islet transplantation is presently 2–4 : 1, in contrast to a ratio of 1 : 1 for whole-pancreas transplantation. This requirement of islets from multiple donors in order to obtain insulin independence is a major obstacle in islet transplantation and limits the number of patients that can benefit from the procedure. In clinical islet transplantation, a large number of islets (>900,000 islet equivalents) is required to achieve normoglycemia [6, 10]. A human pancreas contains 0.3–1.5 × 10^6^ islets per pancreas of which only 30–50% can be isolated using current islet isolation protocols [29]. It is further estimated that only ~65% of human islets are viable following isolation, suggesting that there is a huge margin for improvement [30, 31]. Indeed, successful restoration of insulin independence following allotransplantation of islets from a single donor per recipient has been reported in eight diabetes patients [32]. Stringent donor and recipient inclusion criteria, optimized islet isolation, culture, and implantation together with a modified immunosuppressive protocol were key factors in attaining insulin independence from the single donor. It will be of interest to learn about the long-term follow-up of these patients. Alternative sources of beta-cells have been detailed in several reviews and range from insulin-producing cells derived from adult stem cells or other progenitors to xenografts obtained, for example, from porcine pancreata [33–35].

The challenges that need to be overcome in clinical islet transplantation are mimicked in murine islet transplantation models, although caution must be used when applying insight gained from animal models to patients. In order to reduce the donor-to-recipient ratio in human islet transplantation, lessons learned from animal models are crucial for improving islet isolation and culture techniques, as well as the function and viability of the transplanted islets [36]. Similar to human islet isolation, the procurement rate of mouse islets is far from optimal. It has been estimated that ~2000 islets are present in a mouse pancreas with the typical islet yield being ~10%, or ~200 islets per mouse [37]. The number of islets engrafted in murine islet transplants is usually 300–500 islets resulting in a donor-to-recipient ratio of 1.5–2.5, and of these islets ~60% have been reported to be lost in the peritransplant period [38]. When considered with the finding that only 20% of the pancreas is necessary to maintain normoglycemia following partial pancreatectomy [39], it should be sufficient to transplant fewer islets and still obtain euglycemia as long as the islets are healthy and functional. By increasing the yield of viable islets obtained from each pancreas, it should be possible to decrease the donor-to-recipient ratio.

## 3. Suboptimal Islet Transplantation

In animal models of islet transplantation, a number of approaches have been used to obtain euglycemia following transplantation of a suboptimal islet mass. Strategies to prevent apoptosis and promote immune regulation, revascularization, and reinnervation in addition to providing metabolic rest for the implanted islets can all be beneficial to long-term transplant outcome. Apart from direct administration of cytoprotective agents in the peritransplant period, vehicles used to express these substances include gene therapy, porous scaffolds, and gelatinous microspheres. Gene therapy approaches using lenti-, adeno-, or adenoassociated viral vectors show promise as a means to modify islet protein expression prior to transplantation. The benefits and issues associated with using gene therapy to improve islet survival in the peritransplant period and assist long-term engraftment as well as the risks associated with transfer to a clinical setting have recently been reviewed [40]. 

### 3.1. Strategies to Prevent Apoptosis or Induce Immunomodulation

Beta-cells are highly susceptible to apoptosis triggered by a variety of mechanisms including ER stress, reactive oxygen species, and cytokine-mediated cell death. Readers are referred to several recent in-depth reviews of beta-cell apoptosis [41–45]. Approaches aimed at promoting anti-apoptotic and anti-inflammatory properties in suboptimal islet transplantation include activation of the inducible cytoprotective protein, heme oxygenase-1 (HO-1) by administration of cobaltic protoporphyrin IX to minimize functional impairment, and apoptosis of the transplanted islets [46]. Alternatively, exposure to carbon monoxide, the product of HO-1, generates cytoprotective cyclic GMP and its dependent kinases [47]. Ex vivo gene transfer of A20, an inhibitor of the proinflammatory nuclear factor-*κ*B inhibitor, has been shown to protect islets from cytokine-mediated beta-cell death and activation of the extrinsic caspase cascade [48]. Engraftment of a functional, suboptimal islet mass was maintained up to six months by inhibiting the intrinsic caspase cascade via treating islets before transplant with gelatinous microspheres containing a cell membrane-permeable Bax-inhibiting pentapeptide [49]. This pentapeptide has also been shown to prevent translocation of mitochondrial cytochrome c, which affords further cytoprotection to the islets. Our group has recently demonstrated that overexpression of the X-linked inhibitor of apoptosis protein (XIAP) in islets allows for a suboptimal graft of only 100 islets to normalize blood glucose levels in a syngeneic transplant model [50]. Analysis of the islet grafts in the immediate posttransplant period revealed fewer apoptotic beta-cells in recipients of XIAP-expressing grafts compared to control grafts suggesting that XIAP enhances graft success by inhibiting beta-cell apoptosis. Blocking caspase activation via administration of the pan-caspase inhibitor IDN-6556, which has been used clinically to treat hepatic disorders associated with excessive apoptosis, likewise, results in the successful engraftment of a suboptimal islet mass [51].

### 3.2. Strategies to Induce Revascularization and Reinnervation

Via an intricate network of fenestrated capillaries, islets receive 5–10% of the pancreatic blood volume despite comprising only 1-2% of the pancreatic mass [52]. As a consequence of isolation, islets are severed from their nutritious, normoxic environment and prompt revascularization is crucial to prevent apoptosis and ensure successful islet function after transplantation. It has been reported that levels of vascular endothelial growth factor (VEGF) in transplanted islets are markedly reduced a few days after transplantation and that the time required for revascularization of transplanted islets is approximately two weeks [53, 54]. Hence, several groups have focused on accelerating graft revascularization by stimulating vessel formation using growth factors to minimize the hypoxic and nutrient deprivation of islets in the peritransplant period. Suboptimal islet grafts have in many studies been shown to fare better by transplanting islets transduced with vascular endothelial growth factor (VEGF) or the controlled release of gelatinized fibroblast growth factor (FGF). These two growth factors act to stimulate angiogenesis and de novo vessel formation by facilitating the differentiation of endothelial cells into a vascular plexus [55–58]. A recent study evaluated administration of liver-specific FGF-21 in a suboptimal islet transplantation setting [59]. Apart from stimulating angiogenesis, this growth factor is involved in the regulation of glucose, lipid, bile acids, and phosphate metabolism and was shown to improve the engraftment rate of a minimal number of islets (80 islets). Yet another study took advantage of the synergistic effects of VEGF and hepatocytic growth factor to enhance revascularization and prevent delayed angiogenesis commonly associated with islet transplantation [60]. An alternative approach used to stimulate angiogenesis as well as prevent inflammation involved cotransplantation of allogeneic islets with syngeneic adipose tissue-derived stem cells [61]. The survival and function of these islet grafts are enhanced likely due to increased levels of von Willebrand-positive cells and a decreased infiltration of macrophages and CD4- and CD8-positive cells.

Severing of extracellular matrix proteins during islet isolation causes trauma to the islets, disrupts the islet-matrix interface, and leads to anoikis. In an attempt to facilitate the regeneration of a normal islet microenvironment after transplantation, collagen IV, fibronectin, or laminin was absorbed to porous scaffolds prior to seeding them with 125 islets to enhance integration of the graft [62]. Of the three extracellular matrix proteins tested, collagen IV maximized graft function by accelerating reversal to euglycemia as well as improving the response rate to a glucose challenge suggesting that integrin-mediated interaction is important for islet survival. The authors speculate that the extracellular matrix proteins may lead to upregulation of VEGF, which stimulates both host and donor endothelial cells to promote vascularization. Fibroblast-populated collagen matrix is another novel scaffold used to promote isograft survival [63]. Via production of fibronectin and growth factors, this particular scaffold enhances islet cell viability and function and stimulates islet cell proliferation.

Another important circuit to reestablish promptly after transplantation is islet reinnervation. One study undertook a neurotrophic approach by culturing islets for 48 hours with nerve growth factor, which has been shown to play an important regulatory role in beta-cell function and facilitate reinnervation [64]. This approach resulted in prolonged survival of a suboptimal, syngeneic islet graft by enhancing beta-cell function. Given that larger islets, tend to have a necrotic core following isolation, another study explored the difference between small and large rat islets in a marginal transplantation model [65]. The smaller islets (<150 *μ*m) were superior to larger islets, and the authors speculated that this difference was caused by the faster revascularization of the smaller islets. It is likely that smaller islets also reinnervate faster and hence overall regain metabolic control earlier.

### 3.3. Additional Strategies to Minimize Number of Islets for Transplantation

A report documented that maintaining islets in a pelleted, compacted state prior to transplantation is detrimental to transplant outcome and that immediate engraftment is recommended [66]. Others have demonstrated that treating recipients with insulin implants in the weeks immediately before and after transplantation to counteract hyperglycemia provides a metabolic rest for newly engrafted islets and improves transplant outcomes [38]. Likewise, continuous treatment with the insulinotropic drug liraglutide, a long-acting human glucagon-like peptide 1 analog, improved glucose homeostasis in a syngeneic, suboptimal islet transplantation model [67].

## 4. Perspectives

The approaches discussed in this paper focus on establishing a nurturing microenvironment for successful engraftment of a suboptimal islet mass and substantiates the direct link between number and viability of islets and transplantation outcome. It is likely that adjunct therapies or combination methodologies are necessary to enhance long-term graft survival by targeting several of the pathways that cause primary graft nonfunction, partial loss of the islet graft, or graft rejection. A synergistic approach that prevents beta-cell apoptosis while modulating the immune response and enhancing prompt revascularization seems promising. To this end, proteins that inhibit cell death such as XIAP show promise as a means to lower the donor-to-recipient ratio. Developing alternative gene delivery vehicles that are less toxic and immunogenic will be of importance. The impetus for continuous optimization of the initial islet isolation and transplantation protocol is strong. These advances hold great promise to extend availability of islet transplantation to a much larger group of type 1 diabetes patients. 
